# A unique dedifferentiated tumor of the retroperitoneum

**DOI:** 10.1186/1477-7819-2-25

**Published:** 2004-07-23

**Authors:** Shahzeer Karmali, Halligrimur Benediktson, Walley Temple, Oliver F Bathe

**Affiliations:** 1 Department of Surgery, University of Calgary, Calgary, AB, Canada; 2 Department of Pathology, University of Calgary, Calgary, AB, Canada; 3 Departments of Surgery and Oncology, University of Calgary, Calgary, AB, Canada

## Abstract

**Background:**

Dedifferentiated liposarcomas represent heterogeneous tumors with lipomatous and nonlipomatous elements starkly juxtaposed. It is thought that the high grade nonlipomatous elements of the tumor portend a worse prognosis.

**Case Presentation:**

A 19.8 kg heterogeneous retroperitoneal tumor was successfully and completely resected. Because of its extent, no additional treatment modalities were practicable. The tumor soon recurred. The recurrent tumor differed from the primary tumor in that it was more homogeneous, consisting mainly of nonlipogenic, calcific tissue.

**Conclusions:**

Dedifferentiated liposarcomas are known to have a very high recurrence rate. The biological behavior of dedifferentiated liposarcomas is likely dictated by the most aggressive element of these heterogeneous tumors.

## Background

Sarcomas arising from the retroperitoneum are rare tumors, accounting for 10–15% of all soft tissue sarcomas [1]. Liposarcoma is the single most common soft tissue sarcoma and accounts for at least 20% of all sarcomas in adults [1]. Classification of liposarcoma into four types, based on morphologic features and cytogenic aberrations, is now widely accepted [2]. These four types are (a) well differentiated; (b) dedifferentiated; (c) myxoid/round cell and (d) pleomorphic. The extent of differentiation, as reflected by histological grade, remains the most important determinant of clinical course and of ultimate prognosis for patients with liposarcoma after resection. The following case illustrates the great morphological and biological heterogeneity of these tumors. A very rapid recurrence was observed, and this recurrence was considerably less heterogeneous than the primary tumor, consisting mainly of the calcific, nonlipomatous component.

## Case presentation

**Figure 1:**
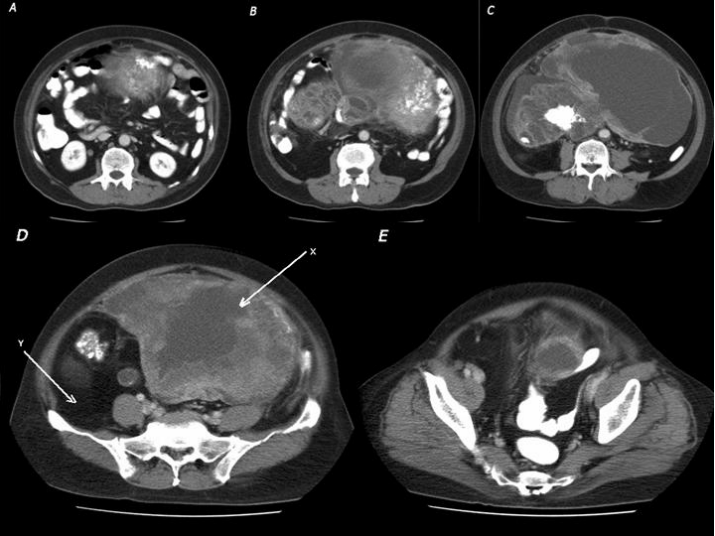
CT appearance at multiple cuts (A – E) of a huge retroperitoneal mass with lipomatous and nonlipomatous components. The nonlipomatous component (arrow X, Panel D) contains calcific elements. Note the posterior extension of the lipomatous component (arrow Y, Panel D), which extends superiorly (Panels A – C). The exact boundaries of this component are difficult to appreciate on CT.

A 65-year-old male presented with a three-week history of progressively worsening abdominal distension. He denied any abdominal pain but stated that he noticed an increased frequency of bowel movements. His past medical history was unremarkable. On examination, he was afebrile and had a hugely distended abdomen with an immobile, nontender mass occupying all four quadrants of the abdomen. Computed tomographic (CT) scan revealed a large, heterogeneous lobulated mass occupying most of the abdomen (Figure 1). The peripheral component appeared lipomatous and the margins of this component were difficult to estimate accurately. There was also a heterogeneous nonlipomatous component that contained areas of lesser density, as well as a central stellate region of calcifications. The preoperative differential diagnosis included a retroperitoneal sarcoma (especially dedifferentiated liposarcoma), desmoid tumor, undifferentiated carcinoma, carcinoid or sclerosing mesenteritis; lymphoma was also considered.

**Figure 2:**
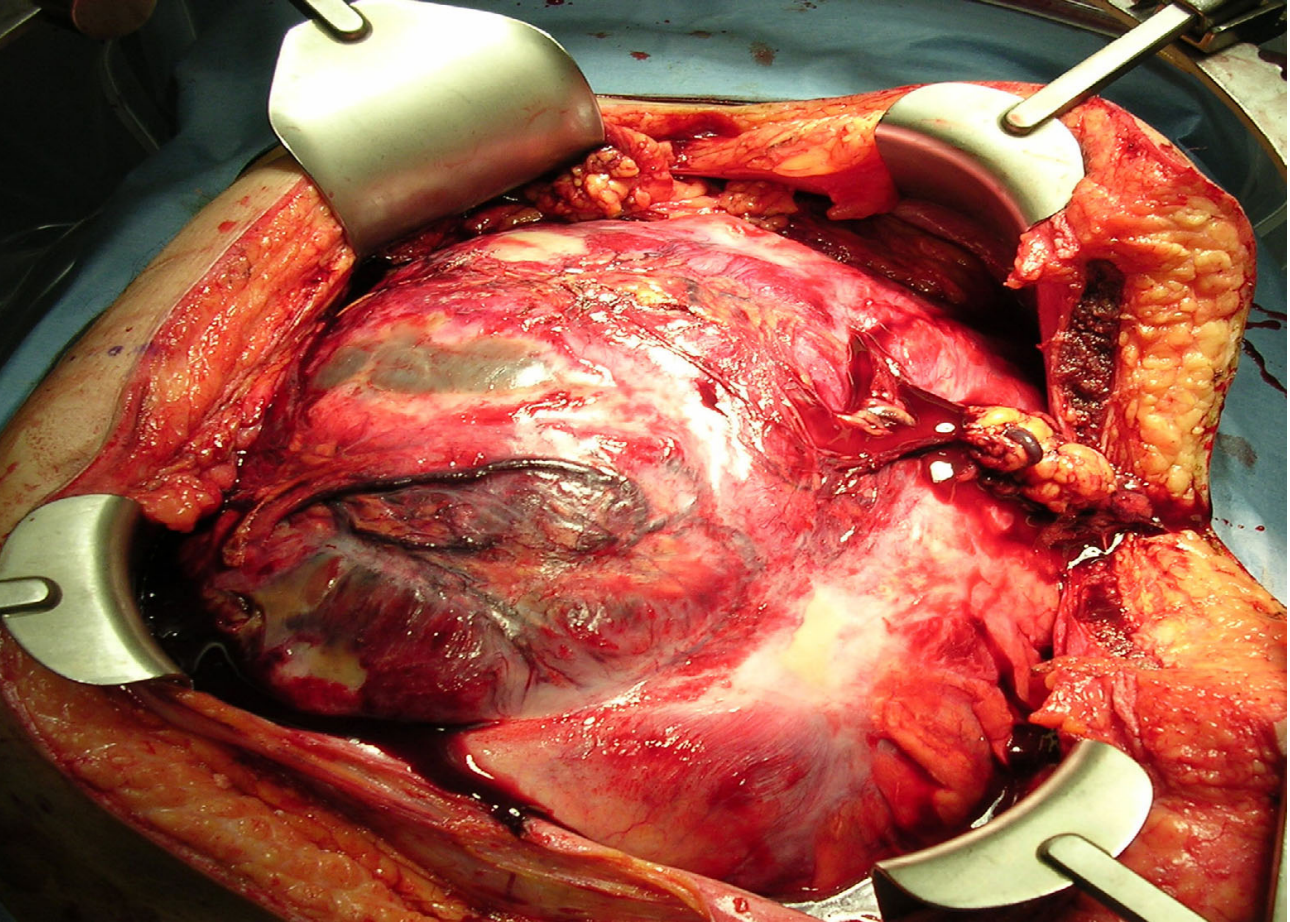
Operative exposure of the tumor through a T-type abdominal incision.

While neoadjuvant chemotherapy and radiation comprise a frequent approach for retroperitoneal sarcomas at our institution, the extent of the tumor made this approach unfeasible. Resection was therefore planned unless an intraoperative biopsy revealed lymphoma. Resection was accomplished through a T-type incision (Figure 2), and entailed removal of the right kidney, terminal ileum, ascending colon, sigmoid colon and the left spermatic cord structures, all of which were intimately attached to the mass. Encasement of the external iliac artery and vein was also encountered near the end of the procedure. This was not fully appreciated preoperatively, as that component of the tumor was so much less conspicuous on CT than the rest of the tumor, given its fatty consistency (Figure 1E). The mass was split in half to facilitate dissection from the iliac vessels. An anastomosis was constructed from descending colon to rectum. A transverse colon mucous fistula and an ileostomy were brought out, as the ileum was dusky at the end of the procedure.

**Figure 3:**
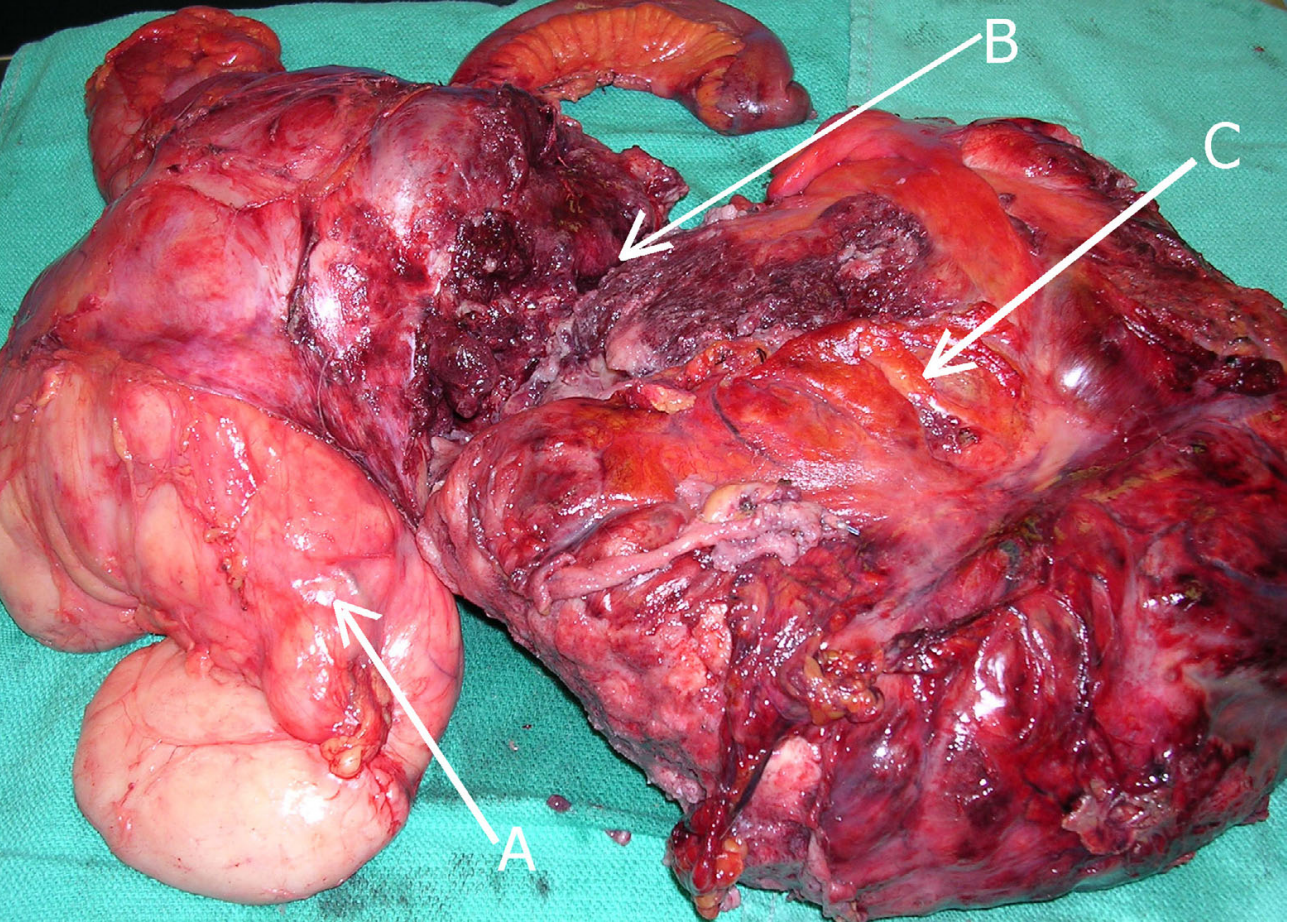
*En bloc *resection specimen of heterogeneous tumor with attached organs. Note the lipomatous regions (A), the calcified areas (B), and the remaining nonlipomatous component (C).

**Figure 4:**
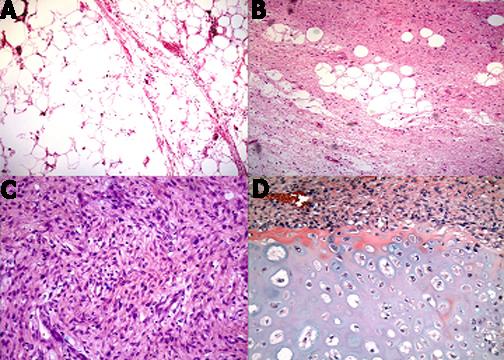
Histologic appearance of various elements of the tumor as sampled in different regions. A. Well differentiated liposarcoma. B. Low grade spindle cell component. C. Cellular spindle cell component. D. Chondrosarcomatous component.

The tumor was submitted for histological examination in two parts measuring, 30.0 × 10.0 × 6.5 cm and 32.0 × 20.0 × 10.0 cm, weighing 19.8 kg in total (Figure 3). On gross examination the mass was variegated, with fleshy and solid cystic degeneration containing areas of osseous consistency. Microscopic examination revealed a juxtaposition of well differentiated liposarcoma and a spindle cell sarcoma with heterologous chondrosarcomatous elements consistent with a dedifferentiated liposarcoma (Figure 4).

**Figure 5:**
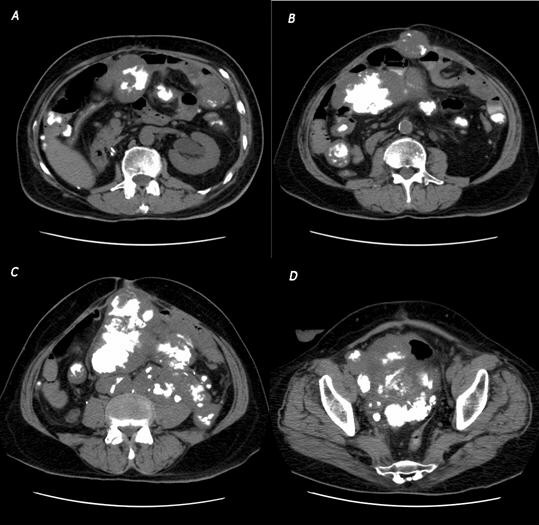
CT appearance at multiple cuts (A – D) of the recurrent tumor. The recurrence was more homogeneous than the primary tumor, consisting almost completely of the calcific component of the nonlipomatous portion of the tumor.

The patient was discharged from hospital on the seventh postoperative day. He was followed in the surgical oncology outpatient clinic monthly. Four months after resection the patient had a follow-up CT scan, which demonstrated an intra-abdominal recurrence consisting almost completely of a calcified, nonlipomatous tumor (Figure 5). The patient died one month later.

## Discussion

The patient in our case manifested the dedifferentiated variant of liposarcoma. The term "dedifferentiated liposarcoma" refers to the development of a high grade nonlipogenic sarcoma juxtaposed to a well differentiated liposarcoma [3]. The majority (80 – 90%) occur primarily *de novo*, although secondary dedifferentiation can occur with multiple recurrences of a well differentiated liposarcoma [4]. CT and Magnetic resonance imaging scans typically reveal well defined nonlipomatous masses associated with fatty tumor; the transition between the two components is characteristically abrupt, although blended transitions are seen in about 20% of cases [5]. Calcifications appear in about 30% and usually correspond to osseus metaplasia, although they may represent osteosarcomatous or chondrosarcomatous elements. The most frequent phenotype of dedifferentiation is that of a high grade pleomorphic malignant fibrous histiocytoma-like sarcoma [4,6]. Other phenotypes observed include leiomyosarcomatous, rhabdomyosarcomatous, osteosarcomatous and angiosarcomatous elements, as well as other nonlipogenic elements [3,7]. A further distinctive pattern in some cases is the presence of micronodular spindle cell whorls, often associated with ossification [8].

Among liposarcomas, the presence of features of the dedifferentiated variant strongly portends a worse prognosis. The overall 5-year survival of dedifferentiated liposarcomas is 20%; the 5-year survival of well differentiated liposarcomas is 83% [9]. Dedifferentiated liposarcomas recur locally in 40 – 83% and distant metastases appear in 15 – 30% [4,7,9]. Therefore, histomorphologic features impact outcomes related to retroperitoneal liposarcomas.

While generally the phenotype of the nonlipogenic component does not impact prognosis of dedifferentiated liposarcomas, the presence of calcifications has been identified as an adverse prognostic factor [5]. In the present situation, it is obvious that the biologically most aggressive component consisted of the calcified (chondrosarcomatous) component. That is, the recurrence was less heterogeneous than the primary tumor, as it had widespread and dense calcifications, but no obvious lipomatous elements.

Complete resection of the tumor is perhaps the most important factor determining long-term survival. Unfortunately, the rate of complete respectability is only about 53% [10]. As illustrated in the present case, in addition to the limitations imposed by the retroperitoneal anatomy, another obstacle to successfully obtaining margins is the difficulty in distinguishing normal retroperitoneal fat from the lipogenic component of the tumor [9]. This was illustrated by the underestimation of the extent of the tumor around the iliac vessels. Moreover, the intraoperative decision to remove the kidney was made in view of the difficulty in distinguishing normal perinephric fat and neoplastic fat; kidney was not involved with tumor, once examined microscopically. Indeed, in a series of retroperitoneal liposarcomas from Memorial Sloan-Kettering Cancer Center, nephrectomy was performed in 38% of patients, although the number in which kidney was actually involved on pathology was not reported [9]. Thus, anatomical constraints and difficulty distinguishing more differentiated fatty tumor from normal fat limit the surgeon's ability to confidently and completely remove all neoplastic elements.

## Conclusions

Dedifferentiated liposarcomas represent aggressive variants of liposarcomas. Each morphological element of these heterogeneous tumors may manifest completely different biology. The overall biological behavior of dedifferentiated liposarcomas is likely dictated by the most aggressive element, which typically resides in the nonlipomatous portion of the tumor.

## Competing Interests

None declared.

## Authors Contributions

SK, HB, WT, and OB made substantial contributions to the intellectual content of the paper, in the interpretation of data, and in drafting the manuscript. All authors read and approved the final manuscript.
